# Agronomic Challenges and Opportunities for Smallholder Terrace Agriculture in Developing Countries

**DOI:** 10.3389/fpls.2017.00331

**Published:** 2017-03-17

**Authors:** Tejendra Chapagain, Manish N. Raizada

**Affiliations:** Department of Plant Agriculture, University of GuelphGuelph, ON, Canada

**Keywords:** terrace agriculture, mechanization, wall crop, legume, agronomy, erosion, Nepal, *conuco*

## Abstract

Improving land productivity is essential to meet increasing food and forage demands in hillside and mountain communities. Tens of millions of smallholder terrace farmers in Asia, Africa, and Latin America who earn $1–2 per day do not have access to peer-reviewed knowledge of best agronomic practices, though they have considerable traditional ecological knowledge. Terrace farmers also lack access to affordable farm tools and inputs required to increase crop yields. The objectives of this review are to highlight the agronomic challenges of terrace farming, and offer innovative, low-cost solutions to intensify terrace agriculture while improving local livelihoods. The article focuses on smallholder farmers in developing nations, with particular reference to Nepal. The challenges of terrace agriculture in these regions include lack of quality land area for agriculture, erosion and loss of soil fertility, low yield, poor access to agricultural inputs and services, lack of mechanization, labor shortages, poverty, and illiteracy. Agronomic strategies that could help address these concerns include intensification of terraces using agro-ecological approaches along with introduction of light-weight, low-cost, and purchasable tools and affordable inputs that enhance productivity and reduce female drudgery. To package, deliver, and share these technologies with remote hillside communities, effective scaling up models are required. One opportunity to enable distribution of these products could be to “piggy-back” onto pre-existing snackfood/cigarette/alcohol distribution networks that are prevalent even in the most remote mountainous regions of the world. Such strategies, practices, and tools could be supported by formalized government policies dedicated to the well-being of terrace farmers and ecosystems, to maintain resiliency at a time of alarming climate change. We hope this review will inform governments, non-governmental organizations, and the private sector to draw attention to this neglected and vulnerable agro-ecosystem in developing countries.

## Introduction

Terrace farming or terracing is a major source of livelihoods for a large section of hillside farmers across the world. In terracing, the hilly or mountainous terrains are divided into narrow but graduated steps, typically 2–3 m wide and 50–80 m long across the slopes, to facilitate growth of field crops, horticultural crops, fodder, and other crops that require specific management practices (e.g., irrigation), alone or in agroforestry systems (Riley et al., 1990; Wymann von Dach et al., 2013). Rice terraces of the Philippine Cordilleras (UNESCO, 2015), *Hani* rice terraces in China (Colinet et al., 2011), *andenes* in the South American Andes (Branch et al., 2007; Goodman-Elgar, 2008), and *conuco* in the West Indies (Watts, 1987) are a few of the well known examples of terrace farming. Terrace farming is one of the most predominant forms of agriculture in Asia and the Pacific (China, India, Nepal, Bhutan, Japan, and the Philippines), South America (Peru, Ecuador, Bolivia), Central America (Mexico, Honduras, Guatemala), Europe (Italy), Middle East (Yemen), and East Africa (Ethiopia, Tanzania, Rwanda); however, there is no reliable quantitative data of the global land area or number of farmers involved in terrace agriculture. In China alone, terraced land is reported to be approximately 13.2 million ha while it is over 2 million ha in Peru (Inbar and Llerena, 2000; Lu et al., 2009).

Terrace farming has been used for centuries. Historical records suggest that terraces have been in practice in Tanzania for about 300–500 years; in Peru, Guatemala, and Mexico for about 2,000 years; in Cyprus for approximately 3,000 years; in China for about 4,500 years; and in Yemen for the past 5,000–6,000 years (Sandor, 2006; Showers, 2006; Engdawork and Bork, 2014). In the Peruvian Andes, the Incas (Branch et al., 2007) and civilizations before them, notably the Huarpa (Leoni, 2006) and Wari (Branch et al., 2007; Williams, 2002), used to harvest potatoes, quinoa and corn from sharp slopes and intermittent waterways. Terraces covered about a million hectares throughout Peru at the height of the Incan civilization in the 1400s and fed a vast empire (Graber, 2011). These terraces and the traditional farming knowledge and expertise were lost over the centuries when the Spanish imposed their own crops and forced the Incas to leave their lands (Graber, 2011). In the West Indies, a system of shifting cultivation known as *conuco* existed in the 14th century for the production of starch and sugar rich foods; and various forms of hunting, fishing, culling, and collecting of wild plants and animals, for fat and protein. The *conuco* system, originally derived from the South American mainland, consisted of planting complex intercrops involving vegetatively propagated crops (e.g., starchy tubers such as cassava and sweet potatoes) in a well-drained situation or in mounds in the wetlands (Watts, 1987, see below in the Opportunities section: *Adoption of the Taino cultivation system*).

Terrace farming has several merits. It is considered one of the oldest and most successful techniques for conserving soil and water during cultivation on steep slopes (Mountjoy and Gliessman, 1988; Kirby, 2000; Bewket, 2007; Engdawork and Bork, 2014). Terracing of slopes conserves soil regardless of the cultivation system used to produce field crops: in Parana (Brazil), it has been shown to reduce runoff and soil losses by half (IAPAR, 1984), while in New Brunswick (Canada), soil losses were dramatically reduced from 20 t ha^−1^ yr^−1^ to 1 t ha^−1^ yr^−1^ (Chow et al., 1999) when terracing was combined with the construction of grass waterways and contour planting of potatoes. In Western Japan, there was less sediment runoff after terracing compared to the practice of planting trees on slopes (Mizuyama et al., 1999). Similarly, in Ecuador and Spain, traditional terrace farming combined with contour cropping reduced erosion compared to non-terraced fields (Inbar and Llerena, 2000), where the role of vegetation cover was found to be more critical than the type of terrace.

In addition, the narrow terraces restrict the use of diesel engines and tractors (Spugnoli and Dainelli, 2013), and as a result farmers use locally made agricultural tools (Tiwari et al., 2004). Furthermore, the remoteness of many terrace farms away from cities restricts access to inputs such as chemical fertilizers and agrochemicals. As a result, terrace farms consume relatively limited fuel, energy, and water (Wymann von Dach et al., 2013), resulting in a low carbon and environmental footprint. Also, cultivation on hillsides and mountains involves use of local and traditional practices for farming (Mountjoy and Gliessman, 1988; Hawtin and Mateo, 1990), and it offers potential for building on the indigenous practices and knowledge of local mountainous environments. The maintenance of traditional knowledge has been shown to help maintain biodiversity and diverse ecosystem services (Riley et al., 1990).

Despite the benefits of terrace farming, there are challenges. Only a subset of terrace farms across the globe have shifted from ancient to modern techniques (Mountjoy and Gliessman, 1988). The majority of terrace farms are managed traditionally using simple tools, limited animal draft power, and relatively abundant household labor (Vogel, 1987; Mountjoy and Gliessman, 1988; Varisco, 1991). Furthermore, the majority of terrace farms are under rainfed conditions and lack irrigation. As a result, many terraces are not as productive as farms that have appropriate mechanization and irrigation.

The current literature regarding terrace farming has focused on estimating soil erosion (Inbar and Llerena, 2000; Londono, 2008), soil and water conservation (Bewket, 2007; Engdawork and Bork, 2014), land use dynamics (Kammerbauer and Ardon, 1999; Gautam et al., 2003), economic benefits and ecological impacts (Liu et al., 2011; Sharda et al., 2015), and sustainability and sensitivity of terrace agricultural systems to climate change (Branch et al., 2007); however, the peer reviewed knowledge of key challenges and appropriate agronomic practices and tools for sustainable intensification of terrace farming has received considerably less or no attention. This article explores the existing agronomic challenges and offers possible opportunities for terrace intensification and livelihood improvement with a focus on smallholder farmers in Nepal and other developing countries.

## Challenges

The challenges associated with smallholder terrace farming to facilitate a shift from traditional subsistence based agriculture to more profitable and less laborious farming are listed below.

### Technical/technological challenges

#### Narrow and limited land for agriculture

As noted above, the chief characteristic of terrace farming systems is the prevalence of narrow terraces making them challenging for agriculture due to limited surface area. As the slope becomes steeper, the terrace becomes narrow, and the height of the terrace wall (risers) increases. A terrace wall that is taller has a greater chance of collapse and requires more maintenance which in turn is more difficult when the terrace is narrow.

Individual farmers in hills and mountains often have widely dispersed terraced fields at different altitudes enabling them to produce a wide range of crops, but the farm holdings tend to be small and fragmented. For example, in Nepal, the average agricultural landholding per household is 0.8 ha (CBS, 2011); the holding in the hilly region is about 0.77 ha, and that number shrinks to 0.68 ha in mountains (Adhikary, 2004).

Opportunities to increase cash income are limited to the crops that are resistant to local conditions, have a good market value, and are easily transported (e.g., low volume, light weight, Hawtin and Mateo, 1990); however, cash sales conversely reduce the food that is available for household consumption (Paudel, 2002). For this reason, there is a need for terrace farmers to intensify production using the entire surface area available.

#### Increased labor/difficult to mechanize the farm operation

The terrace slope and width are two important factors that determine the type and power of machinery used to perform agricultural operations on terraces (Spugnoli and Dainelli, 2013). A narrow terrace (<2 m wide) prevents the use of machinery or animal power. Therefore, the vast majority of farmers in hills and mountains use locally made hand-held agricultural tools (Table 1) appropriate to narrow terraces (Tiwari et al., 2004). In wider terraces (i.e., 2–6 m wide), animate power (humans and draft animals) is a major source of farm power but not machinery (e.g., diesel engines, tractors) which is restricted due to physical constraints (e.g., having little area to turn around the machines; trees or shrubs in the middle of the terraces, Paudyal et al., 2001; Shrestha, 2012). A steeper slope makes the movement of people and tools more challenging—analogous to a staircase having deep steps. The time required to move a machine up and down a terrace (i.e., against gravity) increases labor demands (Paudyal et al., 2001).

**Table 1:** **Locally made tools used in terrace farming in the hills and mountains of South Asia (Images courtesy of Lisa Smith, University of Guelph, can be re-used under the Creative Commons BY license)**.

**Names**	**Purpose and Make**	**Power Source**	**Diagram/Photo**
**TOOLS FOR FIELD PREPARATION**			
Plogh	Ploghing tool made of wood, iron, or steel frame with an attached blade or stick used to cut the earth	Animate (Animal and Human)	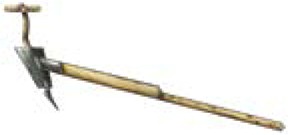
Spade, Kodali	Digging tools made of wood (handle) and a wide sharp tip of metal; a shovel differs from a spade in the form and thickness of the blade	Human	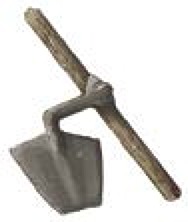
Leveler	The plank of the leveler is made of wood, and the shafts made of bamboo, to level the field after ploghing	Animate (Animal and Human)	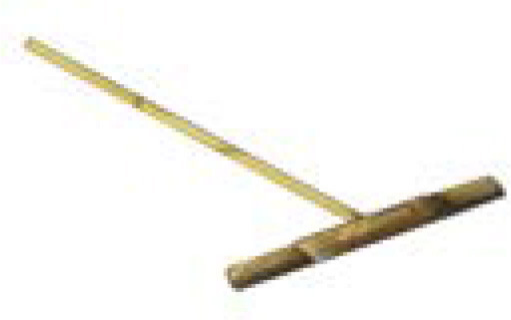
Hammer	The wooden hammer used to break the leftover clods after leveling	Human	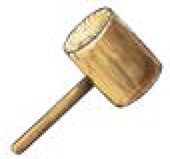
**TOOLS FOR INTERCULTURAL OPERATIONS**			
Rake	Rake is made of wood (handle), and the metal hard tines used to spread around mulch, dirt, or rocks	Human	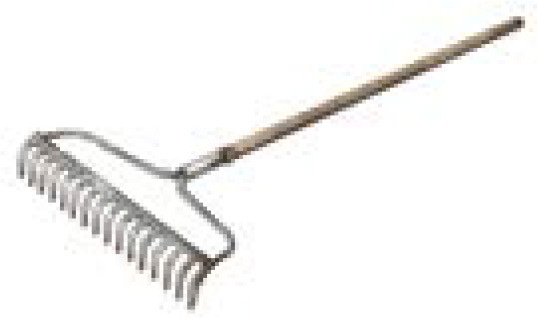
Hand cultivator	Weeding/soil loosening tool for small areas	Human	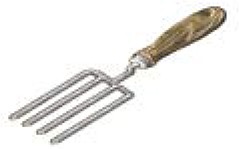
Trowel	Digging tool to make small holes to plant seedling, normally used for transplanting	Human	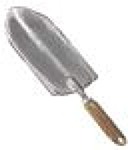
Shovel	Tool to move material from a pie as a scoop, not for digging	Human	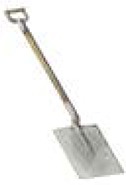
**TOOLS FOR HARVESTING AND POST-HARVEST OPERATIONS**			
Sickle, Hansiya, Karaunti	Cutting/harvesting tools made of wood (handle) and un/serrated curved blade	Human	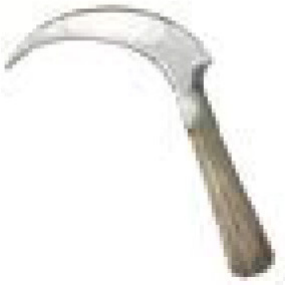
Winnower	Semi/circle structures made of bamboo to separate the grains from husk	Human	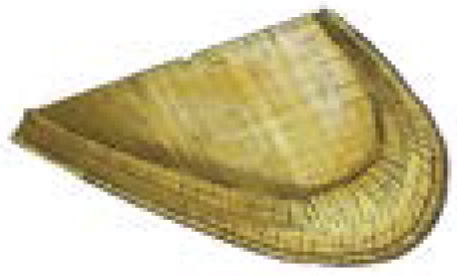
Sieve	Bamboo made tools to separate grains and align materials/dirt	Human	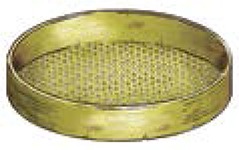
Bamboo Basket	Big bamboo basket (*doko*), and small bamboo basket (*tokari*), used to carry farmyard manure (FYM) and farm produce	Human	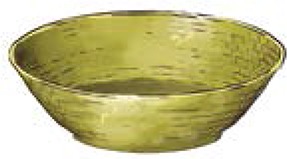
Hand mill	The base (grinder) is made of rock with a wooden/bamboo handle used to grind flour and pulses	Human	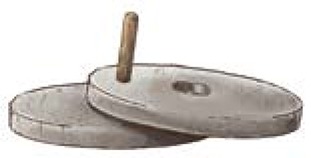
Sac	Made of jute or plastic, used to store the cleaned/processed farm produce	–	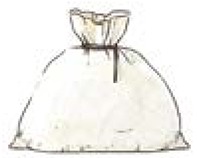

For example, Africa has the lowest farm power base of any region with less than 10% of mechanization services provided by engine-powered sources (Kienzle et al., 2013). At the same time ~25% of farm power is provided by draft animals and over 70% comes from human labor (mostly from women, the elderly, and children). Furthermore, local farmers are aided by only rudimentary tools and equipment for soil preparation, crop care, transport of goods, and bucket irrigation. On terraced lands in Nepal and India, animate power is predominantly used to carry out activities that require more energy and time, such as field preparation, sowing, intercultural activities, and harvesting and post-harvest operations (Shrestha, 2012; Singh, 2014). Use of indigenous bullock-drawn wooden plows for field preparation followed by harrowing with a wooden plank (Table 1) normally consumes more labor, while seed broadcasting, clod-breaking using a wooden-hammer, manual weeding, harvesting using a plain sickle, and threshing (beating with sticks) cause the most drudgery for women, the elderly, and children (Adhikary, 2004; Singh, 2014). Farmers need to walk up and down hillsides with these tools along with bags of seed, seedlings, manure, fertilizers, and the harvest. Men need to guide livestock, while women tend to and/or hold children. Therefore, the development of light weight and less bulky tools for hillside and terrace farms is required to minimize drudgery associated with transportation and field operations.

#### Poor access to agricultural inputs, markets and services

Accessibility and/or remoteness are major issues in terrace farming, especially in developing countries. The difficult topography and low population densities in hills and mountains relative to lowland areas increase the investment and maintenance costs required for basic infrastructure (e.g., roads) to enhance market chains and communication with the outside world (Wymann von Dach et al., 2013).

Widely dispersed terrace fields at different altitudes are not easily accessed from home, and such remoteness further reduces access to markets and urban centers (Adhikary, 2004). Access to improved tools and power machinery is restricted by poor/no electricity in remote regions, while other inputs such as fertilizer, improved seeds, and access to urban markets are limited by the poor road networks (Paudyal et al., 2001; Adhikary, 2004; Spugnoli and Dainelli, 2013). Decreasing numbers of livestock in hills and mountains in recent years has also limited the availability of livestock manure on terrace farms in South Asia (Sharma, 1996; Singh, 1997). This situation, combined with the higher rates of illiteracy and low purchasing power of hillside farmers, further limits access to technological and extension services including access to commodity pricing information. These challenges lead to subsistence livelihoods (Adhikary, 2004).

### Environmental challenges

Soil loss and degradation due to water erosion are major issues for hillside farmers. For example, in Rwanda, crop productivity in the highlands is decreasing as a result of intensive farming on steep slopes which has caused soil loss and declining soil fertility (Clay and Lewis, 1996; Kagabo et al., 2013). Soil losses in the north-western highlands of Rwanda range from 35 t ha^−1^ yr^−1^ to more than 100 t ha^−1^ yr^−1^ depending on the agricultural practices and steepness of the slope (Lewis, 1988). In Ethiopia, annual soil loss from croplands is 35 t ha^−1^ yr^−1^ resulting in a 1–2% annual loss in crop production (Hurni, 1993).

In general, terracing conserves soils compared to non-terraced fields regardless of the cultivation system used to produce field crops. Nevertheless, soil erosion and the loss of topsoil are still major threats to terrace farming. Terracing affects the rate of soil erosion caused by water through its effect on local hydrology, runoff characteristics, soil moisture and soil characteristics (Chow et al., 1999). It is obvious that the effective utilization of terrace lands and maintenance of terrace walls can reduce runoff and soil losses (AAFC, 1999) but terracing also disturbs the soil strata, and considerable soil loss occurs during construction and in the first few years, leading to initial declines in soil fertility (ICIMOD, 1998).

Soil erosion control by terracing is often found to be the most expensive soil conservation practice (Inbar and Llerena, 2000) as it requires tremendous labor and investment for construction and maintenance of the terrace walls. As a result, terrace abandonment and terrace deterioration are observed more often in areas with local labor shortages, which result in massive soil losses (Vogel, 1988; Cerda-Bolinches, 1994; Harden, 1996). Gallart et al. (1994) explained that terraces retain an excess of water leading to saturation, and consequently storm runoff can affect the base of terrace walls due to steepness and sparse vegetation cover (Lasanta et al., 2001; Van Dijk and Bruijnzeel, 2003). Saturation and storm runoff lead to further deterioration of terraces due to gully formation. In Tanzania, bench terracing was found to be inappropriate in areas having thin topsoil as it exposed the infertile subsoil during construction, held excess water, and triggered landslides (Temple, 1972). Soil loss from bench terraces was ~5 t ha^−1^ yr^−1^ under rainfed conditions (Carson, 1992). Terracing increases soil loss if constructed in sandy and coarse textured soils and on very steep slopes (ICIMOD, 1998).

Changes in soil characteristics after terracing degrade soil quality (Hamdan et al., 2000; Li and Lindstrom, 2001) through increased runoff and soil erosion (Ternan et al., 1996). Even within the terrace, soil fertility increases in the lower part of terraces compared to the upper part due to the down slope movement of organic matter and nutrients (Gebremedhin et al., 1999; Walle and Sims, 1999; Dercon et al., 2003; Kagabo et al., 2013). For this reason, it is considered efficient to initially construct small contour ridges made of vegetation and stones compared to the diversion terraces, to entrap sediments and protect soil strata, permitting gradual terrace formation after 4–10 years (Roose, 1986).

### Socio-economic challenges

#### Poverty

Hills and mountains are the least developed areas in most developing countries. A significant land area is covered by mountains and highlands in Mexico (45%), Guatemala (75%), Colombia (40%), Ecuador (65%), Peru (50%) (Mateo and Tapia, 1990), Uganda (19%), Kenya, and Tanzania (23%) (Wymann von Dach et al., 2013), Ethiopia (45%) (Hurni, 1993), and Nepal (76%) (Panth and Gautam, 1990), providing homes for millions of people below the poverty line. People living in hills and mountains are predominantly rural and depend on agriculture and natural resources for their livelihoods, and typically have no alternative source of income or employment. A decline in the $1-a-day poverty rate in rural areas has been reported in East Asia and the Pacific region; however, rural poverty is rising notably in Sub-Saharan Africa and South Asia (World Bank, 2008). Most subsistence farmers cannot afford expensive tools and technologies, and the cost of higher education.

Nepal, for example, lies in 157th place out of 187 countries listed in the UNDP's Human Development Report with a Human Development Index of 0.463 (IFAD, 2015). Over 30% of Nepalese people live on less than US $14 per person, per month (CBS, 2011) with 25% of people living below the poverty line. This figure goes up to 75% in the high hills and mountains where the terrain is rugged, rainfall is low and the land is degraded and difficult to farm. The average land holding in Nepal is 0.8 ha with a population pressure on cultivated land of 6.5 persons ha^−1^ (Panth and Gautam, 1990). There exists a higher concentration of mass poverty, household food insecurity, poor nutrition, unemployment, and illiteracy in these areas (Manandhar, 2014).

#### Labor shortage (human capital)

Labor scarcity associated with the increased permanent migration from hilly regions to nearby cities in search of better paying jobs and quality of life is a major constraint to the management of terrace agriculture (Mountjoy and Gliessman, 1988; Patel et al., 2015; Gartaula et al., 2016). Such labor shortages can lead to terrace abandonment as already noted. In Mexico, for example, the *Cajete* terrace system has been in use since pre-Hispanic times (1000 BC) which involves collection of water in small water reservoirs on the terrace plateaus (Mountjoy and Gliessman, 1988). The use and maintenance of the Cajetes has gradually declined due to rising labor costs as many of the farm families left the farm for higher paying jobs. In China, hillside terracing had been greatly promoted since the early 1950's by the Upper and Middle Yellow River Administrative Bureau for comprehensive erosion and sediment control; however, it appeared to be less effective due to its labor-intensive nature and the relatively low productivity of the terraced plots (Leung, 1996). In Nepal, 93% of farmers face some amount of terrace failure that requires an average of 14 days of labor per year for repair activities (Gerrard and Gardner, 2000). Construction and maintenance of terraces require tremendous labor and investment that keeps every male member of the village busy on their own farm. In such a situation, the migration of male heads of families or their engagement in non-farm occupations often leaves women responsible for terrace maintenance (Reij et al., 1996). Since women are then faced with two jobs, it is difficult for them to pay sufficient attention to repairing terrace walls which leads to further deterioration. In addition, temporary migration sometimes forces migrants to lease or rent land to other farmers or to leave land in the care of immediate relatives—without ownership, these individuals are less likely to maintain the walls (Riley et al., 1990; Leung, 1996; Reij et al., 1996). These arrangements further deteriorate the terrace land since plots are often continuously cropped without manuring, which in turn weakens soil structural stability and leads to increased run-off and soil loss. This situation is further exacerbated when natural calamities such as landslides, drought, fire, hailstorm, and earthquake cause tremendous loss of seeds and biodiversity thus disrupting the immediate growing season and future seasons (Panth and Gautam, 1990; Riley et al., 1990).

#### Illiteracy/cultural barriers

The hill and mountain peoples of developing nations are highly vulnerable as they are associated with concentrated settlements of marginalized groups. In South Asia, these marginalized groups include ethnic, caste, and minority groups, particularly those of the lowest caste (*Dalits*) as well as indigenous peoples (Upreti and Butler, 2014) for whom life is a constant struggle for survival. For example, in Nepal and India, access to food and shelter has been a challenge for the majority of people living in hillside communities as they have large families or have very small landholdings, with high rates of illiteracy (Adhikary, 2004; Bista et al., 2013; Singh, 2014). Women and girls have traditionally been confined to domestic chores and fieldwork, often lack access to resources, education, and employment opportunities, and have lower wages and high vulnerability to domestic violence. Providing assistance to remote and scattered highland communities has been difficult, in addition to communication barriers with people speaking distinctive languages. As a result, households are unaware of modern farming practices and marketing strategies that could increase overall production (beyond subsistence farming) and profit from sales. It is important to note, however, that these farmers do have considerable knowledge passed down over generations, rooted in ecology, agronomy, and biodiversity (e.g., complex rotations and polycultures), and which is adapted and resilient to the local environment.

## Opportunities

Despite the above challenges, there are tremendous opportunities to increase farmers' net return from terrace agriculture compared to conventional hillside systems. Terraces offer a wide range of opportunities to grow a variety of crops, livestock, and forest species alone or in combination. Criteria for selecting crops include those that are adapted to the local context, require low inputs, increase nutrition, and/or income, and promote climate change resiliency, while specific tools and practices should be effective, low cost, scalable, light weight (for tools), reduce female drudgery, and be environmentally friendly by reducing runoff and erosion. Since terracing is mostly practiced in remote hills and mountains, emphasis should also be given to practices that reduce requirements for labor and transportation, and are easy to use to reduce farmer's dependency on service providers and/or institutions. Finally, terrace farmers will undertake new activities primarily if there is a direct and obvious economic benefit from the selected interventions over existing practices (Chan and Fantle-Lepczyk, 2015). Terrace land can be intensified agro-ecologically using one or more of the following ways:

### Terraces for introducing low cost practices and products for eco-friendly farming

Table 2 shows examples of low-cost practices and tools that provide opportunities to intensify terrace cropping systems while improving sustainability and/or drudgery. Terraces offer opportunities to offset agricultural losses related to low and erratic rainfalls in hills and mountains by utilizing the inverse slopes and by adopting soil moisture conservation tillage technologies (e.g., contour ridging, tied-ridging, and mulch-ripping on hillsides or by adopting zero or minimum tillage) in order to increase germination and yields (Vogel et al., 1994; Guto et al., 2012; Chen et al., 2015). In Zimbabwe, no till tied-ridging and ripping into maize residues greatly reduced surface runoff and increased the infiltration rate, resulting in higher grain, and biomass yields due to increased root depth and root length density (Vogel et al., 1994). Ridges and tied-ridges can be constructed using local equipment (e.g., mouldboard plow) that is designed to be animal-drawn. Similarly, the use of plastic film combined with straw mulch in winter wheat increased grain yield (35%) and water use efficiency (25%) compared with conventional practices in the Loess Plateau, China (Chen et al., 2015). The combination of minimum tillage and the living vegetative barriers of the *leucaena* tree (*Leucaena trichandra* Zucc. Urb.) also resulted in reduced competition for water between barriers and companion crops in the water deficient highlands of Kenya (Guto et al., 2012). In this region, yields of maize and soybean were shown to be suppressed by the barrier-crop interface (e.g., due to shading) but the yield losses were consistently compensated by improved crop performance at the center of the terraces. Similarly, in the Anjenie watershed of Ethiopia, terrace farming showed increased yields of maize (1.73 t ha^−1^) and barley (1.86 t ha^−1^) over the control (0.77 and 0.61 t ha^−^^1^ for maize and barley, respectively) as a result of water conservation and erosion control (Adgo et al., 2013), resulting in improved household income and food security.

**Table 2:** **Low-cost and sustainable practices and tools for terrace farms (Source: SAKNepal, **2017**)**.

**Tools or Practices**		**Potential Benefits**
**SPECIFIC PRACTICES**		
1.	Planting wall crops (trailing or climbing types) on vertical slopes	Utilizes unused slopes; ground cover protects soils; improved economic returns.
2.	Planting legumes (hanging or bush type) on terrace edges	Protects edge-collapse; reduces surface runoff; additional yield and biomass from edge crops.
3.	Living grass (napier, vetch, lucerne) barriers	Reduces surface runoff; protects soil from water erosion.
4.	Use of cover crops/dry season legume forages	Protects soil during rainy season and conserves moisture during the dry season; mitigates dry season outmigration.
5.	Micro-climate based diversification	Utilization of niche based micro-climatic pockets provides tremendous opportunities to grow diverse crops of economic value.
6.	Contour ridging	Formation of ridges perpendicular to the slope; prevents runoff; crops are planted on the ridges as well as in the furrows.
7.	Tied ridging	Formation of repeated small earthen ties between the ridges on which crops are planted; accommodates runoff, preventing water erosion.
8.	Mulch ripping	Parallel rips into the soil along with maintaining crop residues/straw mulch (e.g., maize stover) or cover crops on hillsides catches/prevent surface runoff.
9.	*Taino* cultivation	Raising crops in a *conuco*, large mounds created on hillsides, employed principles of conservation farming.
10.	Inverse sloping	Cultivation on terraces (and/or wall base) that are sloped toward the upper wall, not the edge, to promote more efficient capture of moisture and nutrients.
11.	Eco-tourism	Increases the number of tourists and income from tourism.
**OTHER PRACTICES**		
1.	Intercropping (e.g., maize + cowpea, maize + ginger, ginger + soybean, millet + soybean, mustard + pea, wheat + pea)	Increases yield; increases N fixation by legume intercrops; increases N accumulation in soil; reduces pest, disease, and weed problems.
2.	Include high value legumes/vegetables in sequence (relay) combined with plastic house and drip irrigation	Legumes that fit well to the existing cropping sequence increase net income; mitigates dry season outmigration.
3.	FYM preparation under shade, use of terrace gravity flow to collect livestock urine	Improves manure quality (% NPK) and matures earlier than local practice (exposed FYM heap).
4.	Improved variety/seeds of field crops	Increases yield and mature earlier than local varieties.
5.	Planting legume seeds coated with appropriate rhizobia strains/ micronutrients (B + Mo)	Increases nodule numbers; improves N-fixation from atmosphere; enhances crop growth and yield.
6.	Balanced use of chemical fertilizers and organic manure/FYM	Increases yield of the primary crop and the following season's crop; improves soil nutrient content.
7.	Fertilizer micro-dosing	Spot placement of small amounts of fertilizer to seeds/seedlings reduces fertilizer requirements without reduction in grain yield; saves on input cost.
8.	Seed cleaning and treatment before seeding	Increases germination, seedling health and vigor by reducing pests and pathogens.
9.	Zero- or minimum tillage	Growing crops or pasture in hills and mountains with minimal soil disturbance; protects topsoil from wind and water.
10.	*Anabaena*-*Azolla* symbiosis	Adds nitrogen and organic matter to the soil; increases grain yield.
11.	Integrated rice-fish system	Offers complementary use of water and land; improves soil fertility; control aquatic weeds and pest; increase land productivity.
**SPECIFIC TOOLS**		
1.	Jab drill planter	Saves time compared to traditional seed sowing techniques (e.g., behind-the-plogh method); useful for narrow terraces; easy to operate and potentially inexpensive; most effective after initial field preparation (e.g., by mini-tiller).
2.	Mini-tillers	Reduces need for bullocks for field preparation; can be used on narrow terraces; expensive but can be purchased by the community.
3.	Drip irrigation/fertigation	Use of drip-via-gravity provided by terraces to irrigate crops and for applying soluble fertilizers; reduces operation costs and prevents nutrient loss.
**OTHER TOOLS**		
1.	Handheld corn sheller	Inexpensive, easy to use and efficient; requires less effort and reduces drudgery than traditional practices (e.g., beating cobs with sticks).
2.	Fork weeders/farm rakes	More efficient collection of weeds from crop fields planted in rows; reduces backache while weeding.
3.	Electric/gas grain threshers	Requires less time and physical efforts; expensive but can be purchased by the community.
4.	Gloves and knee-pads	Reduces pain in hands and knees during harvesting and intercultural operations; reduces female drudgery.
5.	Low-oxygen grain storage bags	Reduces insect damage; increases seed quality during storage.
6.	Grafting and budding knife	Effective for large scale multiplication of vegetatively propagated fruits (citrus, pear, guava) and fodder trees.
7.	Rain water harvesting structures (tank/pipes, plastic pond)	Beneficial to irrigate high value crops during the prolonged dry season.
8.	Plastic house	Permits pre-season nursery establishment to extend the growing season; facilitates off-season production of vegetables.
9.	Fruit picker	Avoids danger while picking fruits from high branches; provides less or no damage to fruits.
10.	Magnifying glass	Shows magnified image of seeds; helps separate healthy seeds from diseased or damaged seeds.
11.	Manual flour grinder	Hand operated grain mill in remote hills helps prepare flour at home.
12.	Back support belt	Back-brace for lifting heavy equipment + harvest up/down terraces reduces women drudgery.

In high hills and mountains, crops require a longer growing season than low altitudes due to cooler temperatures. Transplanting of vegetative parts (cuttings, tubers, rhizomes) or seedlings from nurseries (e.g., grown in plastic greenhouses) may mitigate this challenge. Plastic greenhouses (i.e., semi-circular to square shaped high tunnels) may also be used to introduce certain high value crops [such as tomato, cucumber, runner bean (*Phaseolus coccineus* L), etc.] by replacing or adding to less profitable field crops. Intercropping (i.e., growing of two or more crops together on the same land) is another opportunity to harvest multiple crops in the same season, increasing land productivity (Chapagain and Riseman, 2012, 2014a,b; Chapagain, 2014) and other ecosystem functions (e.g., nutrient cycling, carbon sequestration, water use efficiency, etc.) in smallholder agriculture (Chapagain and Riseman, 2015; Chapagain, 2016; Thilakarathna et al., 2016). Relay intercropping, where the second crop is seeded after the first crop has reached its reproductive stage but prior to harvesting, also takes advantage of a shorter available growing season. For example, planting of millet, soybean, horsegram, and runner beans before maize is ready for harvest is common in the hills of Nepal (Sharma et al., 2001).

Tools listed in Table 2 are also available on a commercial scale, and they can be procured in Asia at a large scale online such as from Alibaba.com, Indiamart.com, etc. Tools for land preparation (e.g., mini-tillers), sowing (e.g., jab drill planters), weeding (fork and cono weeders), harvesting (corn shellers, millet thresher, etc.), and protective equipment (e.g., knee-pads, gloves) may be effective in reducing drudgery and discomfort, especially for women farmers. Most of the listed tools come at a price ranging from $1–10 which can be purchased by an individual farmer or household; however, a few big machines (such as mini-tillers, electric maize and millet threshers, etc.) may cost up to $500 which can be purchased as a communal-tool by a farmer's group or cooperative and/or at a subsidized price if provisioned by the national government as seen in Nepal (SAKNepal, 2017).

#### Terrace wall and edges to grow cash crops and conservation farming

Table 3 shows a variety of crops such as legumes, vegetables, spices, and flowers that can be planted on terrace walls to cover unused vertical slopes and thus help increase land productivity and economic return. Such crops can either be climbers planted at the base of terrace walls (cucurbit family crops such as gourds, pumpkin, chayote) or waterfall-type crops grown from terrace edges (such as rice bean). Preliminary data has shown that growing chayote, pumpkin, and yam on terrace walls can provide up to $100 USD in additional income per household in Nepal (SAKNepal, 2017). Criteria for climbing wall plants include tolerance to wall-associated shade, ideally drought tolerance under the typical rainfed system, and an ability to fit into the existing cropping system. Certain legumes (such as field pea, common beans) are able to climb because they have specialized structures called tendrils, a type of modified aerial stem.

**Table 3:** **Wall growing crops suitable for terrace base and edges**.

**Crop Type**	**Common Name**	**Scientific Name**	**Uses/Purpose**
Wall Base Crops	Black pepper	*Piper nigrum* L.	Dried fruits used as spice and seasoning.
	Bottle gourds	*Lagenaria siceraria* var. Hispida (Thunb.) H. Hara	Fruit used as vegetable.
	Broom grass	*Thysanolaena maxima* (Roxb.) Kuntze	Flowers are used as cleaning tool or broom; shoots are used as fuel and fodder during lean periods.
	Cardamom	*Elettaria cardamomum* (L.) Maton	Seeds used as highly aromatic spice.
	Chayote	*Sechium edule* (Jacq.) Swartz	Fruit, leaf tip, and tuber used as vegetables; prolific; one plant produces ~250 kg of fruit and 20–25 kg of root/tubers (SAKNepal, 2017).
	Pumpkin	*Cucurbita pepo* L.	Fruit and leaf tips consumed as vegetables; wider leaves cover ground surface.
	Rose	*Rosa* spp.	Mostly used as cut flowers; also used to make oil, water/syrup, and essence.
	Sponge Gourds	*Luffa cyclindrica* L.	Fresh fruits used as vegetable.
	Vetiver	*Chrysopogon zizanioides* L.	Drought and frost tolerant hedge crops; deep, strong and fibrous root system bind with the earth making the underground wall strong; the above ground cover slows or stops surface runoff.
	Yam	*Dioscorea* spp.	Starchy tuber vegetables; shoots cover vertical slopes; one plant produces 10–15 kg of tubers (SAKNepal, 2017).
Wall Edge Crops	Blackgram	*Vigna mungo* (L.) Hepper	Native bean of India, dried and split seeds used as a pulse; leaves and seed husks used as animal feed.
	Cowpea	*Vigna ungiculata* (L.) Walp.	Annual vine/bush type legume; fresh pods and seeds consumed as vegetables; plant biomass used as animal feed.
	Horsegram	*Macrotyloma uniflorum* (Lam.) Verdc.	Annual legume, dried seeds used as a pulse or cattle feed; leaves and seed husks used as animal feed.
	Napier grass	*Pennisetum purpureum* (L.) Schumach.	Perennial tropical grass with low water and nutrient requirements; high biomass production; can be harvested 4–6 times per year.
	Pigeon pea	*Cajanus cajan* (L.) Millsp.	Drought resistant perennial legumes; young seeds are consumed fresh as a vegetable and dried seeds as a pulse; leaves and seed husks used as animal feed.
	Rice bean	*Vigna umbellate* (Thunb.) Ohwi and Ohasi	Warm season annual vine legume with edible beans used as vegetable; prolific.
	Soybean	*Glycine max* (L.) Merr.	Annual legume, fresh pods and seeds consumed as vegetable; seeds also processed for their oil and protein for the animal feed industry.

Terracing can be an effective method to save soil, and effective management of walls and edges can further assist this objective (Wheaton and Monke, 2001). Perennial grasses (e.g., vetiver) and other climbing crops (refer to Table 3) can be planted at the base of the wall, while trailing legumes (e.g., ricebean) and fodder species (e.g., napier grass) are best suited for the terrace edge to conserve soil from erosion (Chapagain and Gurung, 2010). Improved agronomic and soil management practices on walls and edges, including promoting inverse sloping on terraces, may promote more efficient capture of nutrients. In vulnerable areas, there is an opportunity to protect soils from erosion during the transition from dry to wet seasons by planting cover crops (e.g., clover, *Trifolium* spp.; vetch, *Vicia* spp.) or catch crops (quick growing vegetables such as lettuce, *Lactusa sativa* L., or Italian rye grass, *Lolium multiflorum* L., etc.). Cover crops, aside from their soil benefits, can be used as green manures or livestock fodder.

### Terraces as sources of food, feed, and medicine

As a wide variety of crops ranging from small herbs to large trees can be grown on terraces, there may be opportunities to diversify and intensify terrace agriculture. The selection of crop and cropping systems is dependent on farmers' decisions, which are conditioned by multiple drivers such as climate, soil type(s), topography, land holdings, farmers' needs, cultural preferences, availability of agricultural inputs (e.g., seeds, fertilizers, etc.), and local market opportunities (Riley et al., 1990; Chapagain and Good, 2015).

In Nepal, for example, the principal field crops grown on terraces include maize (*Zea mays* L.), rice (*Oryza sativa* L.), and finger millet (*Eleusine coracana* L.), while crops such as wheat (*Triticum aestivum* L.), common bean (*Phaseolus vulgaris* L.), field pea (*Pisum sativum* L.), and underutilized and wild legumes can also be planted depending on the season and farmers' interest (Riley et al., 1990; Wymann von Dach et al., 2013). In addition, several vegetables are also grown in terraces including potato (*Solanum tuberosum* L.), tomato (*Solanum lycopersicum* L.), cucumber (*Cucumis sativus* L.), eggplant (*Solanum melongena* L.), okra (*Abelmoschus esculentus* L.), chile (*Capsicum annuum* L.), bitter gourd (*Momordica charantia* L.), and spices such as ginger (*Zingiber officinale* Roscoe), turmeric (*Curcuma longa* L.), onion (*Allium cepa* L.), garlic (*Allium sativum* L.), and other minor crops. Furthermore, terraces in hills of Nepal and other South Asian countries are sources of a wide variety of medicinal herbs (Table 4, organized from herbs to trees) that reportedly offer health benefits.

**Table 4:** **Common medicinal plants found in the Himalayan region (Source: Manandhar, **1992**)**.

**Common Name**	**Scientific Name**	**Uses/Purpose**
Drymaria	*Drymaria diandra* Blume	Annual herb with slender, smooth stem; juice of plant is applied on forehead to treat headache.
Spiny Amaranth	*Amaranthus spinosus* L.	Annual herb; leaves used as vegetable; a paste of root is applied to treat boils; juice of root used to treat fever.
Hemp/Marijuana	*Cannabis sativa* L.	Annual herb; leaf juice is given to cattle suffering from diarrhea; leaf is mixed with cattle feed.
Creeping Woodsorrel	*Oxalis corniculata* L.	Annual/short-lived perennial herbs; plant juice is applied to treat fresh cuts and wounds.
Common/Stinging Nettle	*Urtica dioica* L.	Herbaceous perennial; fresh leaves used as vegetables; leaf powder used as herbal tea; paste mixed with marble powder applied to set dislocated bone.
Centella	*Centella asiatica* L.	Herbaceous perennial; plant juice is used as tonic early in the morning.
False Goat's Beard	*Astilhe rivularis* Duch.	Perennial herb; rhizomatous flowering plants; juice of root used to treat diarrhea and dysentery.
Bajradanti	*Potentilla fulgens* Wall.	Perennial shrub; root powder used for tooth powder; small piece of root is kept between the jaws to treat toothache.
Indian Braberry	*Berberis aristata* DC.	An erect spiny shrub; decoction of bark is used to treat eye and skin disorders.
Indian Rhododendron	*Melastoma malabathricum* L.	A flowering shrub; plant juice is used to treat cough and cold.
Fire Flame Bush	*Woodfordia fruticosa* (L.) Kurz	A large shrub with spreading branches; flower juice used to treat diarrhea and dysentery.
Butea	*Butea minor* Buch.—Ham. ex Baker	A perennial non-climber shrub; seed powder is used as an anthelmintic medicine.
Castor Oil Plant	*Ridicinus communis* L.	A perennial shrub; flower juice is applied to alleviate cuts and wounds.
Staggerbush	*Lyonia ovalifolia* (Wall.) Drude	A deciduous shrub; paste of tender leaf is applied to treat scabies.
Bayberry	*Myrica esculenta* Buch.—Ham. ex D. Don	An ethno-medicinal tree; juice of bark used to treat dysentery with bloody stool.
Needlewood Tree	*Schima wallichii* (DC). Kortha	An evergreen tree; juice of bark is applied to treat fresh cuts and wounds.
Prickly Ash	*Zanthoxylum armatum* DC.	A deciduous spice tree; paste of bark is applied to treat toothache.
White Cedar/China Berry	*Melia azadirach* L.	A deciduous tree with pesticide and medicinal properties; paste of bark is used as anthelmintic.
Nutgall Tree	*Rhus javanica* L.	A dioecious tree; paste of fruit is used to treat diarrhea and dysentery.

### Adoption of the Taino cultivation system

The Taino were a pre-Columbian farmer society in the Caribbean who developed a sustainable system of hillside agriculture by raising their crops in *conucos*, large mounds created on slopes containing complex intercrops including root crops such as squash, sweet potatoes, and yams, along with maize and other New World crops (Watts, 1987). The *conuco* system employed principles of conservation farming including: ensuring the ground was never left bare in part through the use of perennial intercrops such as cassava; use of twigs/mulches to intercept rainwater; and intercropping with nitrogen fixing legumes such as common bean and peanuts. These strategies apparently permitted some mounds to be productive for up to 20 years. Farmers first set fire to the brush before planting root crops to create more fertile soil. The women then used a type of hoe called a *coa* to transplant cuttings into the earth. This system of shifting cultivation was very well suited to the Caribbean environment as it provided good drainage and reduced erosion (Watts, 1987). Though marginalized, *conuco* farming is still in practice today in the Caribbean mountains, especially in Haiti and the Dominican Republic (Houston, 2005).

### Integrated rice-fish system on terraces

Rice terraces can be integrated with fish farming to optimize resource utilization through the complementary use of water and land (Frei and Becker, 2005). This system uses conventional flooded water management practices to increase productivity, profitability and sustainability (Ahmed and Garnett, 2011). The fish improve soil fertility by increasing the availability of oxygen (aeration) and by depositing nitrogen and phosphorus (Giap et al., 2005; Dugan et al., 2006). Furthermore, farmers employ this method for biological control of rice pests (flies, snails, and other insects), and hence the rice-fish system is regarded as an important element of integrated pest management (IPM) in rice (Berg, 2001; Halwart and Gupta, 2004). Fish act as predators, and help control aquatic weeds and algae that act as hosts for pests and compete with rice for nutrients. Moreover, fish eat the eggs and larvae of disease causing insects (e.g., malaria causing mosquitoes, etc.) and help control water-borne diseases (Matteson, 2000). In turn, rice provides fish with planktonic, periphytic, and benthic food (Mustow, 2002). The water temperature is also maintained by the shading effect of the rice, enabling fish to thrive during hot summer months (Kunda et al., 2008).

### Use of Anabaena-Azolla symbiosis in rice fields

*Azolla* is a highly productive and free floating aquatic fern that fixes atmospheric nitrogen is association with the nitrogen-fixing cyanobiont, *Anabaena azollae*. *Azolla* is able to double its biomass in 2–3 days (Kannaiyan, 1993) and is used as an organic bio-fertilizer in rice fields in Asia, but is much less common in East Africa where there is an opportunity to expand the practice. Temperature and light are the most important factors that influence the growth and efficiency of nitrogen fixation in the *Azolla*-*Anabaena* symbiosis in the tropics (Becking, 1979). Therefore, selection for temperature tolerant and photo-insensitive strains of *Azolla* (e.g., *A. microphylla*) represent opportunities (Kannaiyan and Somporn, 1988). Inoculation into transplanted rice fields with the fresh biomass of *Azolla* fronds (200 kg ha^−1^) or the frond based spore inoculum of *A. microphylla* (2.5 kg ha^−1^) can produce ~15–25 tons of a fairly thick layer of *Azolla* (Kannaiyan and Somporn, 1987; Kannaiyan, 1993). The symbiosis adds nitrogen (40–60 kg ha^−1^) and organic matter to the soil after decomposition, and has been shown to cause a 36–38% higher grain yield compared to a sole rice system (Kannaiyan, 1993). Apart from *Azolla*, use of alternate wetting and drying (AWD) or intermittent irrigation helps improve crop performance, productivity and water-efficient production of rice over conventional flooding in water deficit areas (Chapagain and Yamaji, 2010; Chapagain et al., 2011a,b).

### Utilizing micro-climates for agricultural intensification and diversification

Hills and mountains possess diverse climatic conditions that permit farmers to grow a variety of agronomic and horticultural crops. In Nepal, for example, there are four different agro-ecological zones, which can be exploited to produce off-season vegetables, fruits, and other cash crops throughout the year (Panth and Gautam, 1990). The presence of niche based micro-climatic pockets within each of these agro-climatic regions further provides tremendous opportunities to grow a diversity of food crop, fibers, fruit, medicinal plants, and fodder trees of economic value to that region.

### Making better use of natural slopes

Natural slopes on hills and mountains offer opportunities to take advantage of gravity for creative water capture, irrigation, and livestock urine collection. Besides the construction of tied-ridges, rips, and use of inverse slopes during the dry season, gravity can be utilized to irrigate field crops and to capture urine from penned livestock, and then send the water by PVC pipe or locally-sourced bamboo to FYM/compost pits below, to enrich the nutrient content. In areas equipped with drip irrigation structures, plants can be irrigated with urinated water at no additional cost.

Land topography (e.g., east or west facing slopes to different degrees) in hills and mountains further provides opportunities to produce high value crops on slopes and terraces based on their light and moisture requirements. The direction that a slope faces determines when crops are exposed to sunshine during the day. For example, slopes facing northeast in Nepal have successful citrus cultivation due to the availability of early morning sunshine followed by shade at noon that helps conserve soil moisture, whereas plots facing southwest at the same elevation are devoid of citrus trees (Shrestha et al., 2001). Since soil types differ with the land topography, crops that require different soil, climate, and topography conditions can be produced on hills and terraces. In addition, this situation creates an opportunity to adopt site-specific agroforestry systems (crops, trees, pastures, and livestock together).

### Dry season opportunities

In addition to permanent migration (noted above), in the high hills and mountains of developing countries, there is significant seasonal outmigration of farmers during the dry season following harvesting of the main crops (Patel et al., 2015; Gartaula et al., 2016). Farmers migrate to nearby cities and towns for alternative income opportunities such as from carpentry, house/road/bridge construction, etc. This situation can be minimized by introducing practices that utilize the fallow land for planting forages, along with planting of high value crops (seed, vegetable, cash) combined with water harvesting in the rainy season and drip irrigation (SAKNepal, 2017). Selection of crops and/or varieties with different root architectures (i.e., longer and finer roots, including greater number of tips and branching angle, and a lower shoot:root ratio, Chapagain et al., 2014) and *in situ* moisture conservation practices (ridging, mulching, Watts, 1987) may further help to minimize irrigation requirements during dry periods.

### Tourism

Hillside terraces promote eco-tourism. Rice terraces in the Philippines, China, and Japan are very good examples where communities gain income from eco-tourism. The Rice Terraces of the Philippine Cordilleras and the Hani Rice Terraces in Yuanyang, China, were inscribed on the UNESCO World Heritage List in 1995 and 2013, respectively. The Cordillera terraces were the first-ever property to be included in the cultural landscape category of the World Heritage List which helped to increase the number of tourists and income from tourism. The Hani Terraces were built on mountain slopes ranging from 15 to 75° and provide a typical example of the harmony between people and nature; tourists visit to learn about and photograph the local rice farming and ethnic cultures (Lu, 2015).

## Summary and future perspectives

Hills and mountains in developing countries have traditionally been home to millions of smallholder terrace farmers who are facing climate change and female drudgery. They are the least developed and most remote areas in many countries. Millions of needy households in these areas do not have access to agricultural tools and practices. Adding to the problem is that many interventions introduced by the government and non-government organizations may be expensive, environmentally unsustainable or require female labor (i.e., seeding, mulching, weeding, harvesting, and post-harvest operations), and hence are not scaled up or adopted post-project. In recent years, expanding populations in hills and mountains, land fragmentation, the loss of high quality land, reductions in annual yield increases of major field crops, increasing fertilizer use, and associated transportation costs further created additional pressure on hillside agroecosystems. Unfortunately, these regions, which offer greater food production potential, have not been receiving considerable attention by the global research community.

Table 5 summarizes the key challenges and opportunities in terrace agriculture in developing countries. Loss of productive top soils due to erosion is probably the single most important hurdle. Terracing has enabled farmers to grow crops in otherwise impossible locations with minimal loss of soils; however, widespread clearing of hillside forests for fuel and for agriculture, overgrazing, and loss of diversity have increased the risks of soil erosion in many countries. This challenge can be addressed by using low-cost and sustainable opportunities for ecological intensification and diversification of terraces in hills and mountains. What is lacking is a means to package, deliver, and share these technologies to the world's 1.1 billion subsistence farmers who earn $1–2 per day. We recommend that governments and international agencies working in the agricultural sector should dedicate funds to test innovative tools and practices for terrace farms that should be followed by scaling up of the effective interventions. The products and practices that have been previously validated require an effective scaling up model using both government and private sector networks (seed/input companies and their distribution networks, for example). To enable distribution of these products to rural communities, one opportunity is to “piggy-back” onto pre-existing snackfood/cigarette/alcohol distribution networks that are prevalent even in remote mountainous regions around the developing world.

**Table 5:** **Summary of challenges and opportunities in terrace agriculture**.

**Major Issues**	**Opportunities**
Limited land for intensive agriculture	High value crops and cropping systems on terraces; utilization of vertical slopes (i.e., wall), and edges
Narrow terrace design; difficult to mechanize farm operation	Introduction of light and low cost farm tools
Increased labor and female drudgery	Introduction of tools and practices that reduce female drudgery in agriculture
Poor access to services, inputs and markets	Piggy-backing onto pre-existing snackfood/ alcohol/cigarette distribution networks in rural areas; activities to strengthen market networks, capacity building
Erosion and soil loss	Cover crops, catch crops, mulching, living barriers
Poverty	Introduction of purchasable, low cost ($1–10) technologies
Labor shortage	Labor saving tools and techniques
Illiteracy/cultural barriers	Picture illustrations of best practices and tool use; location specific practices
Low yield and net income	Practices that enhance land productivity and resource use efficiency

Such strategies could be supported by formalized government policies and organizations dedicated to the well-being of terrace farmers and ecosystems. In Italy, for example, there are agricultural policies and economic incentives directed at restoring abandoned or degraded terraces, improving existing terraces as well as building new terraces (Agnoletti et al., 2015) which could be adopted in the developing world. In addition, the 2014–2017 Swiss Agriculture Policy aims to address the needs of mountain family farmers by offering better compensation for public benefits provided by agriculture in mountain regions (e.g., tourism as well as other benefits from well-maintained landscapes, Wymann von Dach et al., 2013). In recent years, the urgency of maintaining and improving terrace agriculture has been highlighted and become an important concern of the United Nations, in agencies such as UNESCO (United Nations Educational, Scientific, and Cultural Organization), FAO (the Food and Agriculture Organization), and GIAHS (Globally Important Agricultural Heritage System) (Agnoletti et al., 2015). Such concerns along with the associated policies, practices, and tools that promote the livelihoods of terrace farmers will help to maintain generations of knowledge about mountain ecosystems including the diversity of crops that can be cultivated and collected to maintain resiliency at a time of alarming climate change.

## Author contributions

Author TC designed the study, managed the literature review and wrote the manuscript. Author MR edited the manuscript and assisted in all phases of this review. Both authors have read and approved the final manuscript.

### Conflict of interest statement

The authors declare that the research was conducted in the absence of any commercial or financial relationships that could be construed as a potential conflict of interest.
